# 

**Published:** 1876-07

**Authors:** 


### TABTE OF CONTENTS.

ORIGINAL COMMUNICATIONS—

"Old and New Remedies," by W. F. Barr, M.D., Abingdon, Va................ 385
Sulphide of Calcium, by T. Curtis Smith, M.D., Middleport, O................ 391
Firwein by Dr. A. K. Alley, Atlanta, Ga........................................ 394
Case of Abnormal Fœtation, by John Coston, M.D.......................... : 396
Chloride of Soda in Cancerous Ulcers, by G. M. Rivers, M.D.................. 397

SELECTIONS—

Persesqui-nitrate of Iron in Chronic Diarrhœa, by Kerr, M.D., Galt .......... 399
Diphtheria........................................................................ 402
Headaches of the Decline of Life.................................................. 406
Jaboradi in Cholera Infantum and Scarlatina .............. ..................... 408
Kentucky Shower of Flesh.......................................................... 409
Digitalis in Pneumonia, and Salicin in the Hemorrhage of Typhoid Fever..... 410

EXTRACTS AND GLEANINGS—

Post Nasal Catarrh, 411. Salicylic Acid in Diptheria, and in Rheumatisn, 413. Treatment of Placenta Prœvia, 414. Treatment of Constipation and Diarrhœa with Water and Tincture of Camphor, 415 Treatment of Cleft Palate without Operation, 415. On the Rational Treatment of Common Tape-worm, 416. A Cure for Drunkenness, 416. Iodoform, 417. Employment of Aspiration in Intestinal Obstruction, 417. Treatment of Severe Sprains, 418. Dangers of Absorption of Carbolic Acid, 418. Quinetum, 419. Ulcerative Stomatitis, 419. A New Method of Administering Enamata of Chloral, 419. Leptandra as a Purgative, 420. Gonorrhœal Rheumatism, 420. Psoriasis—Tar Internally, 421. Poisoning by Santonin, and its Relief, 421. Prophylactic in Cholera Infantum, 422. The Abortive Treatment of Dysentery, 423. Treatment of Constipation and Diarrhœa, 423. Treatment of Acute and Chronic Rheumatism by Means of Hot Packing, 424. Curious Incompatibility, 424. Writing and Reading, 425. Use of Cold in Fever, 425. New Cautery, 425. Select Formulæ—Cinch Quinine, Strychnia and Iron, 426. Camphorated Tincture of Opium, 426. New Remedy for Burns—Salicylic Acid, 426. Rules in Administering Arsenic, 427. Rapid Dilatation of Female Urethra, 427. Therapeutic Action of Chlorate of Potash, 427. Chloral, 428. Effect of Extreme Cold on Mind and Body, 428. Chloroform in Dyspepsia, 428. Grindelia Robusta—Its Medical Value, 429. Enuresis in Childhood, 429. Liquid Glue, 429. Pruritus, 430. Stoughton Bitters, 430. Prevention of Bed Sores by Sheet Zinc, 430. Death from Septic Inoculation, 430. Inflamed Testicle, 431. Iodoform Pencils, 431. Falling Hair, 431. Suppression of Urine, 431. Anti-Rheumatic Mixture, 432. Puritus Ani and Vulvæ, 432. Uterine Tonic, Nervine and Alterative, 432. For Dysmenorrhœa, 432. Chafing, 432. Uterine Tonic and Alterative, 433. Marshall Hull's Nerve Tonic, 433. Creosote in Diarrhœa, 433 Simple Amenorrhœa, 433. For Hysteria and Dysmenorrhœa, 433. A Third Denition at the Age Seventy-three, 434. Surgical Anæsthesia in Children, 434. A Precautionary Measure in Cauterizing the Throat, 434. Water-proof Varnish for Paper, &c., 434. The Treatment of Erysipelas, 436. Action of Borax as an Antiseptic, 436. Salicylic Acid, 436. To Cement Wood and Iron, 436. Experiments, 436. Legislation Against Quack Medicines, 436. Balard, 436. The Use of the Decimal Systems, 436. Tonsillitis, 436. The Making of Doctors, 435. Nestle's Mother's Milk, 435. A New Mucilage, 435. Cement, 435. A Convenient Local Anodyne, 435. Teething Infants, 435.

EDITORIAL AND MISCELLANEOUS—

Our Advertising Department...................................................... 437
Atlanta Medical College.......................................................... 437
Centennial—American Medical Association and Convention of Medical Colleges........................................................................ 439
American Medical Association..................................................... 443
Dr. Sims and the Ethics.......................................................... 445

